# Vehicle Cooperative Network Model Based on Hypergraph in Vehicular Fog Computing

**DOI:** 10.3390/s20082269

**Published:** 2020-04-16

**Authors:** Maoli Ran, Xiangyu Bai

**Affiliations:** Inner Mongolia A.R. Key Laboratory of Wireless Networking and Mobile Computing, College of Computer Science-College of Software, Inner Mongolia University, Hohhot 010020, China; 31709033@mail.imu.edu.cn

**Keywords:** vehicular fog computing, vehicular cooperation network, super-degree distribution, cooperation mode of vehicles

## Abstract

In this paper, we propose an optimization framework of vehicular fog computing and a cooperation vehicular network model. We aim to improve the performance of vehicular fog computing and solve the problem that the data of the vehicle collaborative network is difficult to obtain. This paper applies the hypergraph theory to study the underlying structure, considering the social characteristics of the vehicles and vehicle communication. Since the vehicles join the network in accordance with the Poisson process law, the model is analyzed by using Poisson stochastic process and mean field theory. This paper uses MATLAB to simulate the evolution process of cooperative networks. The results show that the vehicle’s super-degree in vehicular fog computing has scale-free characteristics. Through this model, the vehicle cooperation situation can be analyzed, and the vehicle dynamics can be accurately predicted to further improve the performance of vehicular fog computing. The model can be transformed into some complex network models by adjusting the parameters. It has strong universality and has certain reference significance for the research on the related characteristics of VANETs and the theoretical research of the cooperative network.

## 1. Introduction

VANETs is considered to be an important part of the future intelligent transportation system [1], they support a variety of mobile services, from content sharing (such as advertising and entertainment) to information dissemination services (such as emergency operations, natural disasters and terrorist attacks). The Internet of Vehicles ensures driving safety by exchanging valuable information, improving traffic efficiency, and improving traffic conditions [2]. Over the past decade, with the advent of more advanced devices and technologies such as cellular networks and cloud computing [3], automotive networking and related applications have grown significantly. With the development of Internet technology, more and more vehicular applications [4], such as augmented reality (AR) [5], automatic driving, etc. involve complex data processing and storage operations, which require a higher level of data communication, computing, and storage. This poses a huge challenge to existing traditional vehicle networks, especially in terms of communication and computing resources [6].

In order to meet this growing demand for communication and computing, scholars have proposed to introduce advanced technologies, such as cloud computing and cellular networks, into the VANETs. Using the vehicle cloud as a data center and enhanced processing resources is an attractive idea [7]. In the traditional cloud computing-based network structure, these data need to be uploaded from the vehicle to the cloud via a wireless network, and the cloud performs data processing and analysis before returning the processing results to the vehicle. Data upload processing will increase the transmission delay of the task. The influx of massive data will inevitably lead to queuing of services, which will lead to higher network delays. For vehicles that are using autonomous driving in the Internet of Vehicles, high latency is likely to cause serious traffic accidents. Cellular network can provide limited enhanced communication, mainly controlled by network operators, which is inefficient from the application point of view [8]. Mobile cloud computing can bring rich computing resources to mobile users [9], but it is costly and time consuming to upload real-time information [10].

Fog computing can effectively solve the above problems, by deploying a fog server with storage and computing near the user to receive and process user data [11]. The deployment location of the fog server is closer to the user, which can save a lot of data transmission time. The deployment of fog computing nodes in the connected vehicle network has become a popular trend in the research of VANETs. We finally proposed a novel concept of vehicular fog computing (VFC), which uses vehicles as infrastructure to make full use of vehicular communication and computing resources. Specifically, VFC is an architecture that has cloud-based features and provides data, computing, storage and application services to end users. VFC is also differentiated from other existing technologies by being close to end users, and having dense geographical distribution and support for mobility; its bottom layer consists of cooperative vehicles, so that the vehicle is both a fog computing provider and a vehicular fog computing user. It can use computing resources nearby and reduce the use of communication resources. In the fog computing architecture, vehicles and mobile devices are end users, and there is a fog server layer between these edge users and the cloud. The low-level equipment of vehicular fog computing vehicles has high-speed moving characteristics. The stability of the network formed between vehicles needs further research [12]. Therefore, a collaborative and distributed computing architecture that uses vehicles as fog computing resource nodes is first introduced. This architecture makes full use of the resources of fog nodes and collaboration between vehicles. Due to the highly dynamic of the Internet of Vehicles, it is necessary to study the cooperative relationship between the Internet of Vehicles [13]. Studying the cooperative relationship between vehicles can make the architecture provide a better solution for offloading decision-making in vehicular fog computing, further increase the extension of VFC, and improve the performance of VFC.

Vehicular fog computing is a potential application of fog computing in the transportation industry. Vehicular fog computing must not only achieve vehicle road collaboration, but also achieve five-dimensional high-level collaboration of “people vehicle road network fog”. In terms of people, with MaaS (Mobility as a Service) as the core, we provide consumers with one-stop travel services and let consumers become free people [14]. In terms of vehicles, the future vehicles are not only data transmission and reception; Fang is still a computing node and a data sharing node. Smart vehicles will become smarter and smarter; in terms of roads, they will have all kinds of communication methods (LTE, 5G, LTE-V2X, 5G NR-V2X, etc.) Collection and release of roadside traffic information, with local edge computing capabilities, etc., create a smart road through integrated roadside intelligent facilities. In terms of network, C-V2X and DSRC are the two core communication technologies of the Internet of Vehicles [15]; namely mobile edge computing and network slicing will build a flexible network. In terms of fog, an integrated open data public service platform and cloud control platform will be constructed, and at the same time, powerful fog computing will be formed through the collaboration of fog nodes.

This paper first introduces a collaborative and distributed computing architecture that uses vehicles as fog computing resource nodes. This architecture makes full use of the resources of fog nodes, including vehicles and RSU, and collaboration between vehicles, using vehicular collaboration to share computing resources and reduce the use of communication resources [15]. The vehicle-based infrastructure of vehicle-based fog computing has high-speed mobile characteristics [16]. Therefore, the stability of the network formed between vehicles needs further research.

A cooperative and distributed computing architecture based on the vehicle as a fog computing resource node is introduced [17]. This architecture leverages the resources of the fog node and the collaboration between vehicles. Studying vehicle collaboration enables this architecture to make better offloading decisions [18] in fog computing, to better achieve vehicular fog computing performance. The main contributions of this paper are as follows:

- A vehicular fog computing architecture that considers the sociality of the vehicle is proposed. This architecture makes full use of the idle resources of the vehicle.
- The vehicle cooperation model in the bottom layer of vehicular fog computing architecture is proposed. The current fog computing architecture has a common problem, that is, a large part of the performance depends on the accuracy of predicting the behavior of the mobile fog node. This model solves the data problem, which is difficult to obtain data of the vehicle cooperation relationship, and provides reference for the research on the importance and trust relationship of the vehicle network. Most important, we provide a basis for vehicular fog computing aggregation resources and reallocation of resources.

The rest of paper is organized as follows. After presenting the architecture of vehicular fog computing in Section 2, we describe the overview of vehicle cooperative model in vehicular fog computing in Section 3. Then we analyze the characteristics of cooperative vehicular network in Section 4. We conclude the paper in Section 5.

## 2. Related Work

Some studies have been performed to leverage vehicle resources as major providers of roadside services in various domains and interests [19]. In [20], the authors considered how to coordinate computation resources in both the cloud and smart vehicles to solve the task-scheduling problem in the Vehicular Cloud. The authors of [21] put forward a feasible solution that enables offloading for real-time traffic management in fog-based IoV systems, aiming to minimize the average response time for events reported by vehicles. Utilizing the idle resources of parked vehicles reduces the communication service delay of the vehicle connected network effectively, and a solution capable of offloading real-time traffic management is proposed to minimize the average response time of vehicle incident reports. Through the networking of parked vehicles as edge nodes, a distributed urban traffic management system is constructed, which reduces the data transmission pressure of the backhaul link and effectively reduces network delay.

The authors of [22] propose a collaborative task offloading and output transmission mechanism to guarantee low latency as well as the application- level performance. However, in various applications of the future vehicle connected networks, users are extremely sensitive to delay requirements. For applications such as collision warning and departure warning, if the task is migrated to the base station, the task migration method in which the base station performs the task and then returns the results will inevitably increase the network delay [23]. Although a hybrid communication solution that combines V2V and V2I functionality will inherently provide the most efficient and powerful solution, purely infrastructure-based communication will not limit the application level of data distribution and processing to a specific centralized architecture. In fact, centralized architecture will inevitably lead to scalability issues, so V2I infrastructure does not necessarily use centralized architecture. To cope with the information needs of thousands of vehicles in large cities, although multiple distributed subsystems can be deployed to achieve better scalability, a fully decentralized peer-to-peer (P2P) approach is more attractive. Reference [24] introduces the D4V architecture, based on opportunistic mechanisms for the dissemination of data generated by vehicle sensors and drivers. This architecture relies on a peer-to-peer (P2P) overlay scheme. The Cooperative Intelligent Transportation System (C-ITS) mainly uses vehicles and passengers in a certain range, and roadside pedestrians and roadside infrastructure, to improve the extension and service quality of the intelligent transportation system. Research applications include collision avoidance [25], emergency vehicle warning systems [26], traffic mitigation [27], and criminal vehicle surveillance investigations [28]. The information content under consideration can have local validity, that is, spatial range utility, and its explicit lifetime, that is, its time range validity [29]. Reference [30] summarizes the research on the telematics of vehicles based on smartphones in the past ten years, focusing on the use of the mobility of smartphones and sensors on vehicles to achieve friendly and cooperative applications of vehicles. In addition, we focused on the differences between traditional and smartphone-based car navigation, and conducted surveys based on the latest technology classifications in smartphone-based transportation modes, vehicle self-organizing networks, cloud computing, driver classification and road-condition monitoring. Future developments are expected to be driven by advances in sensor technology, evidence of the social benefits of current implementation, and the establishment of assessments of sensor fusion and vehicle behavior. Wahlström, J. et al. proposes a task migration method, using parked vehicles to reduce the network delay; the parked vehicle cannot provide a durable power output because its engine is in a stopped state [31]. With the use of a parked vehicle as a relay node, the network deployment method can improve network stability. Although the current research on fog computing and connected vehicle has become a relatively popular research direction, the literature combining the two is rare [32]. Further research is needed.

Some researchers have also begun to adopt advanced technologies or strategies to address security mechanisms in industrial cooperation. Ai, Z. et al. considering that potential attacking opportunities triggered by this progress are severely impacting the security fortress of current networks, especially in the edge access part, propose a Multi-dimensional Fine-grained Control (MFC) framework to strengthen safety and reliability in Radio Access Networks (RANs) [33]. An identifier mapping mechanism was designed to achieve network isolation, and the MFC framework is established to describe the model structure and implementation processes. Reference [34] found that the receiving buffer of artificial intelligence in the control area would also limit the throughput problem and proposed an Intelligent Collaborative Automation (SCA) scheme, to improve resource usage and overcome buffer limitations. The program provides comprehensive policy options by simplifying comparisons to provide details. Baldi, S. et al. found that, in the existing adaptive oil drainage strategy, two aspects that need to be properly designed are ignored: the two-way interaction between vehicles may cause the stability of the strings to decrease, and the engine saturation constraint will cause the loss of cohesion [35]. The authors propose a novel adaptive platooning strategy handling these two crucial aspects. Uncertain heterogeneous rows should be homogenized to the reference dynamics. Engine constraints are addressed through suggested mechanisms, and this reference dynamics is “less demanding” by properly saturating their effects. The saturation action will prevent all vehicles in the row from reaching their engine limits, thereby maintaining the regularity of the vehicle’s operation and making it more regular.

Therefore, by analyzing and sorting out the current work, we find that most vehicle fog computing solutions face a single technology, with limited performance improvements and vehicle resource utilization needs to be improved. Faced with different scenarios, the high-speed mobility of the Internet of Vehicles and the complex and changing network environment have brought more uncertainty to the Internet of Vehicles. How to effectively improve the extension of vehicle fog computing and the utilization of vehicle resources has become an urgent problem and key question.

## 3. The Architecture of Vehicular Fog Computing

Fog computing [36] involves many technologies, such as resource management, privacy protection [37], network connection, etc. The development of fog computing depends on the continuous advancement of these technologies, and architecture is the cornerstone of all technologies [38]. Many experts and scholars have carried out research on the architecture of fog computing [39]. Existing fog computing architecture research can generally be divided into two categories: cloud-fog-terminal three-level architecture and fog-terminal two-level architecture [40]. The former consists of a cloud layer, a fog layer and a terminal layer. The fog layer provides temporary storage and real-time analysis of the collected data and sends data to the cloud layer on a regular basis. The latter consists of a fog layer and a terminal layer. Cloud servers participate in cooperation with each other, to provide various services, such as distributed vehicle navigation, intelligent traffic lights and local content distribution.

Fog computing is an extension of cloud computing. The fog-based radio access network is proposed to solve the large-scale collaborative signal processing of cloud-based radio access network (C-RAN) architecture, with large delays and no separation of services and control. The fog-based radio access network (F-RAN) was developed on the basis of C-RAN and Heterogeneous cloud-based radio access networks (H-CRAN) and their architectures are basically similar. The main difference is that F-RAN uses software-defined network technology, edge buffer technology, cooperative radio signal processing technology and cooperative radio. Fog computing access points have evolved into management points, and multiple wireless resource management technologies have been sunk into the edge network. This allows business traffic to be transferred from the cloud server to local network equipment, reducing the burden on the fronthaul link, and also reducing delays in small business processing. Adjacent fog nodes communicate via D2D mode or relay mode, to improve spectrum efficiency. Because the communication delay between the end-to-end communication distance is positively correlated, the fog computing usually only considers the two hops between the fog nodes.

The F-RAN architecture integrates wireless network technology, software-defined network technology, social networking and network virtualization computing into fog computing [41]. It is a service processing solution provided in mobile networks close to users. Many features are similar to the MEC model. It has the advantages of distributed deployment, low latency, high bandwidth, high privacy and high security. However, the difference between the two is also obvious. Edge computing mainly uses edge devices near the user, such as edge servers, to share part of the processing tasks. Fog computing can also use fog nodes to cooperate and communicate with each other, to form an edge distributed network. The server shares part of the processing tasks, and it can also use a nearby fog node to take part of the processing tasks. Fog computing is very suitable for distributed computing with a large amount of computing and extremely low latency requirements, which can effectively save server resources, and at the same time, share idle resources of fog nodes and improve resource utilization of the entire environment

F-RAN research is in the early stages of research. Although there are some key technologies to support it, there are still some issues that need to be resolved, such as the choice of transmission mode based on mobile speed, communication distance, location, etc. There are serious interference problems; software-defined networking technologies, social awareness, computing offloading and network virtualization are still immature, and there are even many computer technology that cannot be directly applied to F-RAN. The F-RAN architecture also needs to be further improved. For example, resource classification needs a perfect algorithm, and resource sharing and offloading mechanisms need a perfect mechanism.

In [42], a fog computing platform was developed, to extend the cloud computing paradigm to the edge of the machine-to-machine network, to support the internet of things. The proposed four-layer fog computing architecture supports the quick response at neighborhood-wide, communitywide, and city-wide levels, providing high computing performance and intelligence in future smart cities.

Vehicular fog computing is an effective fusion of fog computing technology and vehicle networking to efficiently provide computing, storage, and processing functions close to the user, so as to meet the application needs of rapidly developing compute-intensive services. Aiming at the combination of these two types of technologies, the overall network framework design has become the primary problem to be solved for the large-scale application of fog computing in the Internet of Vehicles.

When fog computing is integrated with the Internet of Vehicles, fog computing technology can be used to process business data of vehicle users in real time, making it extremely suitable for servicing high-speed vehicles. The entire network framework can be roughly divided into three layers, from bottom to top: the user layer, the fog computing layer and the cloud computing layer. The vehicle user is responsible for data collection and preprocessing at the lowest user level and only makes decisions at the vehicle level; the middle layer is the fog computing layer, which is mainly responsible for real-time data fusion and localization processing, and undertakes decision-making tasks at the regional level; and the cloud computing layer at the core of the network has long been committed to network data development and macro analysis, and plays a role in network-level decision-making. In particular, roadside units distributed at different locations on the road can be upgraded through hardware upgrades and can become a fog node that makes localized decisions for nearby vehicle users. By deploying and making full use of these fog nodes that are widely distributed around vehicle users, the fog computing-based vehicle networking edge computing solution is also suitable for real-time communication management for smart city scenarios; it greatly reduces the network burden of the traditional vehicle networking core network, while significantly reducing the response delay in the vehicle communication process. In order to improve the performance of vehicular fog computing and minimize the real-time response time of the vehicle networking system, we further studied the vehicular fog computing architecture in our previous research. We integrated the advantages and disadvantages of different edge computing technologies, and based on the focus on edge cloud collaboration, we proposed an edge computing framework for the Internet of Vehicles that integrates a comprehensive sense of collaboration between different network layers and different edge servers.

**Figure 1:**
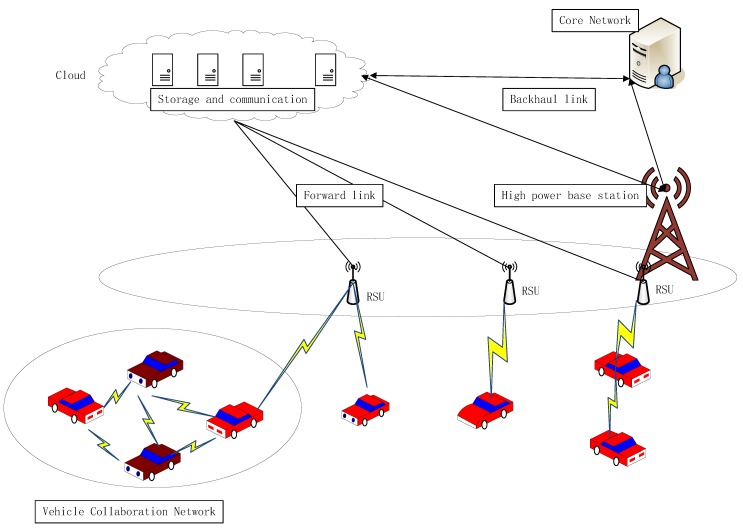
The architecture of vehicular fog computing.

From Figure 1, it can be seen that, when the vehicle needs assistance, the vehicle will send out a help signal. If there are several facilities around, the infrastructure will assist the vehicle to complete the task; if there is no roadside infrastructure around the vehicle, you can pass the surrounding vehicles, to assist in completing the corresponding tasks. Vehicle assistance can be selected based on the vehicle’s historical assistance data. As shown in Figure 1, the architecture is divided into the following layers.

The cloud services layer is uniformly deployed at the top level of the architecture. It is similar to a traditional cloud computing center. It centralizes most application services in the Internet of Vehicles. The cloud service center includes a cloud data center and a cloud composed of high-performance server clusters. The control center can store and analyze a large amount of data collected by various terminal devices. It can also provide users with comprehensive and high-quality services through the deployment of the control center. It is mainly composed of a data center. It uses software-defined networking technology, network virtualization technology and server virtualization technology, to form the data center and integrate computing, storage and other important resources into a “resource pool” to achieve unified management and provide cloud computing services. In this architecture, the cloud can be viewed as a computing entity that improves overall network performance. It is mainly used to improve the quality and computing power of connected car services. It is responsible for handling applications with insensitive delays and large resource requirements. Because data need to be transmitted frequently when in use, it has the characteristics of high latency and high cost. Therefore, this design mainly uses a fog cluster layer and a cloud service layer. Tasks are uploaded to the cloud service layer through the fog cluster layer only when they require a lot of resources and latency is not sensitive.

The fog cluster layer in the vehicular fog computing architecture is mainly composed of two parts, namely fog service equipment and infrastructure. The fog service equipment part is mainly composed of core fog equipment, coordinated fog server, and candidate fog server. The other part of the infrastructure mainly includes roadside node units, communication base stations, cameras, etc. With the advancement of sensor technology, various sensors for road conditions and real-time environmental monitoring have also been applied to the Internet of Vehicles. It extends computing, storage and communication resources to the edge of the network through fog servers deployed on roadside units. Vehicles in the fog service layer access the cloud service layer through roadside units or base stations. The fog cluster layer can respond to and process user requests directly, instead of forwarding all received requests to the cloud service layer. This can offload most business traffic to the widely deployed fog cluster layer, greatly reducing communication resources and processing time. Distributed fog clusters are distributed in various geographical areas and connected to cloud services. The fog cluster layer can be configured with appropriate amounts of computing, storage, and communication resources, and cooperate with each other to handle relatively large tasks. Distributed fog clusters have the following advantages: (1) They can offload most of the data processing to the fog cluster, reducing the link traffic; (2) the fog clusters are connected through a cooperative local controller, which can realize data sharing and mutual assistance; (3) users can obtain corresponding services from nearby fog clusters, which greatly reduces delay and improves service quality; and (4) the fog cluster preprocesses the data uploaded by the fog service layer, which greatly reduces the data volume and relieves the network data load.

In order to further improve the performance and resource sharing of on-board fog computing, a self-organized fog service layer is innovatively proposed. This fog service layer serves users by concentrating idle resources of surrounding vehicles, eliminating the boundary between the terminals and cloud computing services. This is the most flexible and innovative organization, which makes vehicles both providers of “cloud” services and consumers of “cloud” services. The fog service layer is mainly composed of vehicle cloud and users’ mobile phones, computers and other equipment. Vehicle cloud refers to applying the concept of cloud to the traditional Internet of Vehicles. According to a certain principle (within a certain radius, a certain block, etc.), the vehicle group is divided into different sets. Forming a network with vehicle communication and sharing computing resources, storage resources, and spectrum resources are conducive to improving efficiency. The vehicle cloud is not fixed. The members in the collection will change as the vehicle moves, so they can be regarded as mobile cloud nodes. The control center can designate some vehicles as candidate cloud controllers, or set up separate regional cloud controllers in combination to control the use of resources in the vehicle cloud and the entrance and exit of nodes. The fog service layer mainly provides services such as collaborative computing and resource sharing. It can also offload tasks through local communication networks, such as Wi-Fi, Bluetooth, D2D and 802.11p, and rely on the cooperation of surrounding vehicles to process large amounts of data [43], as well as share the computing, storage and other resources of the vehicular fog computing vehicle through the communication network. The sharing vehicle nodes are equivalent to a small data center. When other users request certain resources, they can use it locally, without having to visit a remote data center, thereby greatly reducing latency.

Compared with the traditional architecture, the new architecture proposed in this paper brings many unique advantages to the in-vehicle network. The following points are emphasized here.

Heterogeneous network integration: Through network virtualization and abstraction, all vehicles, roadside units, and roadside infrastructure in the car network can be treated as SDN equipment and managed, using a unified interface, which significantly improves the heterogeneity of VANTs [44].

Mobility: SDN technology can adaptively deploy routing protocols and adjust its parameters according to the rapidly changing external environment. The computational pressure of the cloud service layer can be shared to a certain extent through the deployment of the control plane. This will greatly improve the service quality of high-speed moving vehicles and is more suitable for mobile sensor networks compared to traditional network architectures.

Multi-source data acquisition: With the expansion of scale, there are more and more types of information in the Internet of Vehicles, including traffic detection data, spectrum utilization and parking lot usage. These data need to be dynamically updated, and the collaborative fog service layer in the architecture of this article is very good to meet this characteristic, each core fog server collects the corresponding information through the connected infrastructure and processes it. After an application sends a request message, the cooperative fog server can control one or more core fog servers to respond, so it can greatly reduce the burden on the target server.

Distributed computing and storage: In actual scenarios, the distribution of vehicles is usually random and, in most cases, uneven. When the vehicle density in a certain area is high, the computing and storage pressure of the core fog service equipment responsible for the area will increase sharply. In this architecture, by coordinating the control role of the fog server, the regional network pressure is shared to the core fog servers with less vehicle density in the surrounding area, so as to achieve a distributed working mode and improve network resource utilization.

Multi-path data transmission: The communication of a large number of mobile devices in the Internet of Vehicles places a great burden on the cloud data center and the fog server. Many automotive multimedia have high requirements for data streams, often causing bandwidth congestion. This architecture provides the possibility to alleviate this situation. With the cooperation of the cloud control center and the coordination fog server, the data flow of the same application part can be arranged to be multiplexed, and then transmitted to the destination vehicle through the vehicle cloud V2V communication. It is actually a mode that rationally utilizes the idle bandwidth of other servers and can improve the quality of service.

As can be seen from the above, the architecture of this article provides many new features for the Internet of Vehicles. They all improve the flexibility, mobile support, and scalability of the Internet of Vehicles to varying degrees, and they bring many potentials for the Internet of Vehicles. The application scheme is shown in Table 1, below.

The architecture proposed is an open and layered new architecture, based on the trinity of communication, computing and storage. Computing is used to solve communication technologies, and communication networks are used to carry computing tasks. Cloud computing and fog clusters reasonably integrate computing and communication. This architecture makes full use of a large amount of idle resources on the vehicle and greatly improves the performance of fog computing. However, this architecture and the current fog computing architecture have a common problem, that is, a large part of the performance depends on the accuracy of predicting the behavior of the mobile fog node, so the research on the cooperative relationship between vehicles is very urgent.

We know that no single paradigm can be a total solution. Communication costs, delays and poor network infrastructure are the main obstacles to using new technologies in the connected car to provide sophisticated driving assistance. So far, fog is an unparalleled candidate because it supports high-speed mobility, latency sensitivity and geographical distribution, low cost and lightweight distributed nodes. Therefore, we modified the vehicular fog computing architecture to complete the computing ecosystem and try to solve problems, such as high availability and low latency.

## 4. Description of Vehicle Assistance Network Evolution Model Based on Hypergraph

Among the many complex networks, cooperative networks are popular among researchers, due to their universality in the real world, such as corporate cooperation networks, industry-university-research cooperation networks, actor cooperation networks, scientific research cooperation networks, etc. [45]. A lot of research has been done on network model construction and feature analysis. But most of them are based on the topology of ordinary graphs. The nodes in the graph represent different individuals in the system, and the relationships between individuals are represented by edges. One edge of a normal graph can only connect two nodes and cannot describe many features of the real network. Some cooperative networks are described by bipartite graphs. However, the two types of nodes in the bipartite graphs are heterogeneous and cannot effectively analyze the network’s connectivity, degree distribution and other topological features. With the continuous expansion of the network scale, the types of nodes are diversified, and the relationship between nodes is more complicated. Network system problems that surpass ordinary networks have arisen. Therefore, scholars have tried to apply the principles of super-networks to complex networks. There are two main types of super-networks: hypergraph-based super-networks and network-based super-networks [46]. The superstructure of a hypergraph-based network is a hypergraph, which pays more attention to the dynamic evolution process, so it can be used as a tool for studying the dynamic analysis of complex networks. Hypergraph theory can not only guarantee the homogeneity of points and edges, but also clearly express the relationship between nodes and nodes, and nodes and edges.

The super-network studied in this paper belongs to the hypergraph based on hypergraph [47]. The super-edge in the hypergraph can connect two or more nodes [48]. Any network that can be represented by a hypergraph is a super-network, the definition of a hypergraph:

Let $V=\{v_{1},v_{2},v_{3},v_{4},\ldots ,v_{n}\}$ (*n* is a positive integer) be a finite set.

(1) $e_{i}\neq \phi (i=1,2,3,\ldots ,m)$

(2) $U_{i=1}^{m}e_{i}=E$

The binary relationship H = (*E*,*V*) is called a hypergraph [49]. The elements v1,v2,v3,v4,…,vn of *V* are called vertices of the hypergraph, $E=\{e_{1},e_{2},e_{3},e_{4},\ldots ,e_{m}\}$ are the edge sets of the hypergraph, and the set $e_{i}=\{e_{1},e_{2},e_{3},e_{4},\ldots ,e_{m}\}$ (*i* = 1, 2, 3...*m*) are super-edges of the hypergraph. A vertex belongs to two or more super-edges, which is called super-edge adjacency. The vertex, *v_k_*, belongs to the super-edge number, which is called the super-degree of the vertex, *v_k_*, and is represented by $d_{H}(v_{k})$. If the two vertices belong to the same super-edge, then the two vertices are said to be adjacent. If the intersection of the two super-edges is not empty, the two super-edges are said to be adjacent [50]. Common representations are graphical and matrix forms, including the matrix, adjacency matrix and Laplacian matrix [51].

In the super-network, the nodes in the super-edge adopt the full connection mode, and the node degree is the same as the node degree definition of the conventional network, that is, the number of edges connected in the entire network. The super-degree of the node, that is, the number of super-edges where the node is located, the point over-degree is more meaningful in the real network. Therefore, the analysis of the evolution model focuses on the node overdistribution. In general, a larger node is more important means that the status in the entire network is more important. The super-degree distribution $P_{H}(d_{H})$ of the node is the proportion of nodes with the degree of $d_{H}$ occupying the nodes in the whole network:

(1) $$P_{H}(d_{H})=\frac{N_{d_{H}}}{N}$$

where $N_{d_{H}}$ represents the number of nodes with a degree of $d_{H}$, and *N* represents the total number of network nodes.

Therefore, the analysis and evolution model of this paper focuses on analyzing the distribution of node super-degrees. At the same time, the hypergraph-based super-network has the characteristics of a super-edge connecting multiple nodes, which makes it well described for complex systems with multi-node connections [52]. The evolution of the super-network can also be used to analyze the topological and evolutionary rules of complex systems with multi-node structures. Therefore, this section presents a dynamic evolution model of a vehicle-based fog computing node based on hypergraph. Table 1 outlines a list of the main notations to be used throughout this paper.

Assume that the initial join of the collaborative network is some cooperation nodes with a small number of m0, and the following rules are executed at regular intervals, to expand the network. The symbols and meanings used below are as follows.

(1) Network initialization

Assume that there are m0 cooperation vehicles in the initial network, and jointly complete a certain cooperation task to form a super-edge.

(2) Network growth

With the passage of time, *k*1 new vehicles are added to the previously formed vehicle cooperative network, at regular intervals, to form m super-edges, together with *k*2 existing vehicles in the previous period, and there are m cooperative tasks. The rules added are as follows.

The *k*1 vehicles are added together with the probability *p_i_* to form the super-edge. Some existing network evolution models assume that the nodes are discretely entering the network at the same time interval to form the super-edge, but in the real network, the nodes joining the network are not equal time intervals. There are relatively many nodes added in some time periods, so the nodes are described in terms of the growth model of the network according to the Poisson process. The arrival process of the node satisfies the Poisson process of the constant, *λ*_1_.

(2) $$p_{i}=P\{X=k1\}=\frac{{\left(\lambda_{1}t\right)}^{k1}e^{-\lambda_{1}t}}{k1!}(k1=2,3,4,\cdots )$$

The *k*1 new vehicle nodes select *k*2 (*k*2 < m0) vehicles in the preexisting network with probability, *p_j_*, to form m super-edges at time, *t*, and there is no identical super-edge, *p_j_* satisfy $\sum_{j=0}^{m}p_{j}=1$.

(3) Network connections

In vehicle cooperative network, vehicles that can cooperate are geographically adjacent, and vehicles meet at a certain time. The more times a node works effectively, the higher the probability that it can collaborate with other nodes, to complete a cooperative task (forming a super-edge). The greater the number of vehicle cooperation, the higher the probability that it can cooperate with other vehicles, to complete a cooperative task.

Therefore, when a vehicle cooperative task is added, the newly added vehicle in the cooperation task and the vehicle, i, that has previously joined the network firstly encounter the node with higher activity with probability $f_{i}^{1}$, and the probability of selecting the vehicle and connecting $f_{i}^{1}$ is approximated as the effective activity of vehicle, i, the expression is as follows:

(3) $$f_{i}^{1}=\frac{d_{H}(i)+\alpha }{\sum_{j}\left(d_{H}(j)+\alpha \right)}$$

where $f_{i}^{1}$ is the probability that the vehicle, i, is about to join a task by the super-degree; $d_{H}(i)$ is the super-degree of the vehicle *i*, the number of tasks added; *α* represents the harmonic parameter; and $\sum_{j}d_{H}(j)$ is the sum of all vehicles in the entire network, the sum of the number of tasks participating in all vehicles.

Considering the particularity of the VANETs, when the vehicles need to cooperate, they must also consider the communication range. Therefore, the probability of performing the preferential connection should also consider that some will be randomly connected, so the probability of the vehicle, *i*, being connected in the network is as follows:

(4) $$f_{i}^{2}=\varphi \frac{d_{H}(i)+\alpha }{\sum_{j}\left(d_{H}(j)+\alpha \right)}+(1-\varphi )\frac{1}{n(t)}$$

where n(t) is the total number of nodes in the network at time, *t*.

The probability that the vehicle, *i*, in the network is selected to be connected is $f_{i}^{2}$ and on the right side of the equal sign in Equation (4) indicates that the greater the vehicle super-degree is, the greater the probability of being connected by the new super-edge. The second right side of the equal sign in Equation (4) represents the probability of a random connection.

## 5. Model Analysis

The stochastic process theory and the mean field method are used to theoretically analyze the evolution law of the vehicular fog computing cooperative network to obtain the super-degree distribution of the vehicles. For ease of analysis, $t_{i}$ is the time when the *i*-th batch of vehicles enters the network, $d_{H}(i)$ is the super-degree of the *i*-node at time *t*.

Assuming that $d_{H}(j)$ is a continuous real-valued variable, the average field method is used to know that the dynamic equation of vehicle *j* should be satisfied.

(5) $$\frac{\partial (d_{H}(j))}{\partial t}=\lambda_{1}mk_{2}f_{i}^{2}$$

where *m* is due to the generation of m super-edges, *k*2 is the old node in *k*2 networks in the process of forming the super-edge, the existing vehicle *i* may be formed into the first super-edge, the second super-edge, … *m* super-side selection.

When *t* is large enough, $m_{0}\ll \lambda_{1}tm(k+k1)$, $m_{0}\ll \lambda t$, we can obtain the following:

(6) $$\frac{\partial d_{H}}{\partial t}\approx \frac{\varphi k2(d_{H}(i)+\alpha )}{t(k+k1)}+\frac{\lambda_{1}mk2(1-\varphi )}{t}$$

After simplifying all terms, we can obtain the following:

(7) $$d_{H}(t)=(m+\frac{\left(1-\varphi )m(k1+k2\right)}{\varphi }){\left(\frac{t}{t_{1}}\right)}^{\left(\frac{\varphi k2}{k1+k2}\right)}-\frac{\left(1-\varphi )m(k1+k2\right)}{\varphi }$$

Since the new joining process of the network node satisfies the Poisson distribution with the constant *λ*1, and the addition of the super-edge in the super-network is in accordance with the random process, the instantaneous average super-distribution distribution of the network satisfies the following equation.

(8) $$P_{H}(d_{H})\approx \frac{k1+k2}{mk2\varphi +(1-\varphi )mk2(k1+k2)}{\left(\frac{m+\frac{\left(1-\varphi )m(k1+k2\right)}{\varphi }}{d_{H}(t)+\frac{\left(1-\varphi )m(k1+k2\right)}{\varphi }}\right)}^{\left(\frac{k1+k2}{\varphi k2}+1\right)}$$

It can be seen from the expression of super-degree distribution that the vehicle cooperative model has both the characteristics of random networks and scale-free networks. The strength of the characteristics depends on the preferred connection probability, *φ*, and the random connection probability, 1-*φ*, of the vehicles.

When *φ* = 0, it means that the vehicles are all randomly connected during the network growth process, forming a random network, and the average super-degree distribution of vehicle conforms to the exponential distribution.

When *φ* = 1, it means that the nodes are all optimally connected during the network growth process, forming a scale-free network, and the average super-degree distribution is in accordance with the power law distribution.

**Figure 2:**
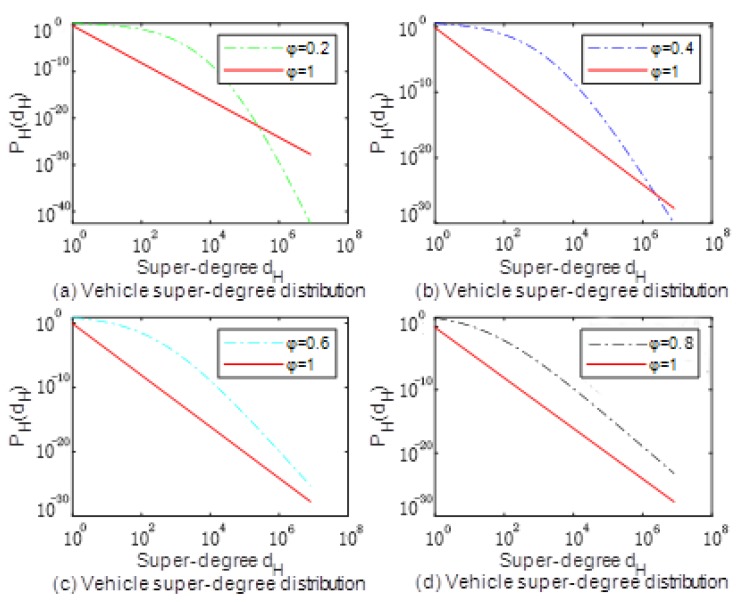
The characteristics with different *φ*.

In order to better understand the nature of the network, we performed numerical simulation analysis on the formed over-degree distribution. At *t* = 1000, *m* = 3, *k*1 = 4, *k*2 = 2, *φ* = 0.2, *φ* = 0.4, *φ* = 0.6 and *φ* = 0.8 respectively, a comparison with *φ* = 1 super-degrees distributed in double logarithmic coordinates is shown in Figure 2.

It can be seen from Figure 2 that the characteristics of the network presented by *φ* = 1 are those of the standard scale-free network. The degree distribution is a power-law distribution, and the characteristics of the network represented by *φ* = 0 are standard random networks, whose degree distribution is bell-shaped distribution. As the value of *φ* decreases, scale-free network characteristics begin to weaken, and random network characteristics begin to appear. As can be seen from Figure 2a, *φ* = 0.2, the network exhibits the characteristics of a random network, and the scale-free characteristic is weak. It can be seen from Figure 2b,c that, when *φ* is relatively close to 0.5, the characteristics of the network are not so obvious. In fact, it is a combination of exponential distribution and power law distribution. In fact, the distribution law of many real networks is difficult to fit or predict with a single distribution function, but a certain limited combination of multiple distributions. It can be seen from Figure 2d that, when *φ* approaches 1, the network exhibits a power-law distribution.

**Figure 3:**
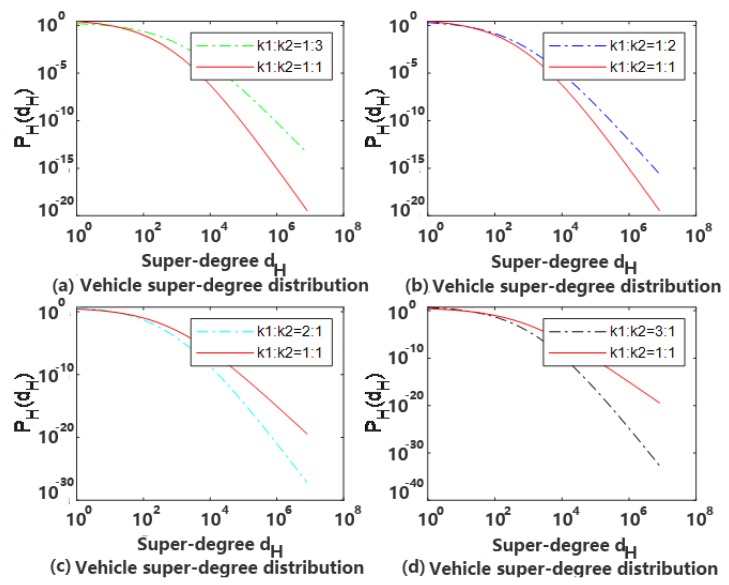
The characteristics with different *k*1 : *k*2.

At *t* = 1000, *m* = 3, *φ* = 0.5 and *k*1: *k*2 = 1/3, 1/2, 2 and 3 respectively a comparison with *k*1:*k*2 = 1 super-degrees distributed in double logarithmic coordinates are shown in Figure 3.

It can be seen from Figure 3 that, when *φ* = 0.5, the probability of random connection and preferred connection in the network is equal, and the characteristics displayed by the network should be similar to the dotted lines in Figure 2b,c. As *k*1/*k*2 is worth changing, the nature of the network presentation is slightly different. The larger the value of *k*1/*k*2 is, the more nodes are newly added to the network, and the vast majority of nodes can only be connected randomly, so the network exhibits random network characteristics.

**Figure 4:**
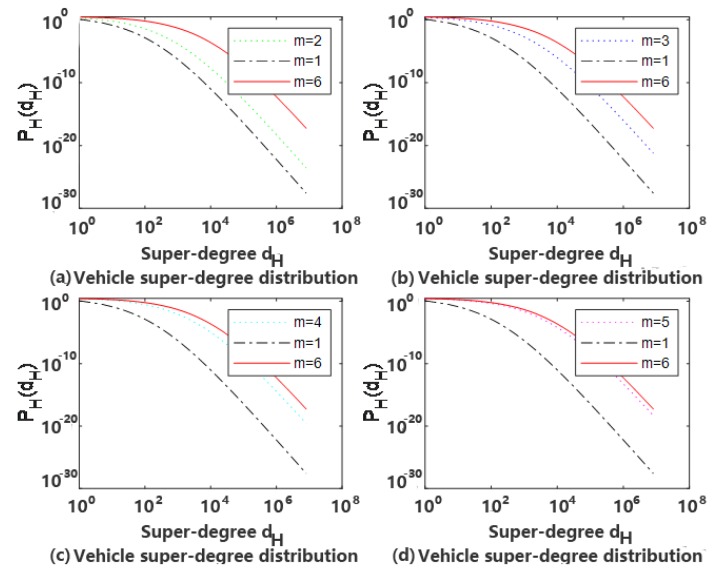
The characteristics with different *m*.

At *t* = 1000, *φ* = 0.5, *k*1 = *k*2 = 3, *m* = 2, 3, 4 and 5, respectively, comparison with *m* = 1 and 2 super-degrees distributed in double logarithmic coordinates are shown in Figure 4.

It can be seen from Figure 4 that, when *φ*, *k*1 and *k*2 are certain, the properties of the network presentation are the same, but the scale-free characteristics are different, but they all have the same power law. The larger *m* is, the more super-edges are formed in a certain period of time, and the over-degree value of the non-scaling characteristics of the nodes in the super-degree distribution is larger. When a network node is added in a certain time in the network, the more nodes that are needed to form a super-edge, the more obvious the characteristics of the network will be in the place where the node is too large, but all have the same power law.

From the above analysis, we can know that the characteristics of the vehicle collaboration network for vehicular fog computing are affected by many aspects, mainly by the network connection mechanism and network growth mechanism.

Traditional vehicular fog computing only considers offloading tasks to roadside infrastructure for processing, and roadside infrastructure is currently not deployed on a large scale, due to its high cost, which cannot be guaranteed to be available everywhere. Because of this, we let vehicle nodes with a lot of idle resources act as fog nodes, to improve resource utilization and offloading coverage. This model can predict the behavior of unloaded nodes from time to space, to expand vehicles. This not only broadens the extension of vehicular fog computing, but also improves resource utilization.

Computing and storage capabilities of vehicular fog computing sinks to the user’s surroundings. As the user is closer to the user, the user’s request no longer needs to be processed through a long transmission network to the distant core network. The fog server offloads part of the traffic and directly processes and responds to users, so the communication delay will be greatly reduced. The latency-saving feature of the fog server is particularly evident in latency-sensitive-related applications such as video transmission and VR. Take video transmission as an example. In the traditional vehicular fog computing method, when each user terminal initiates a video content call request, it first needs to pass through the roadside unit and then access it through the base station, and then connect to the target content through the core network, and then layer by layer. By performing backhaul and finally completing the interaction between the terminal and the target content, it is conceivable that such a connection and layer-by-layer acquisition method is very time consuming. After introducing the vehicular fog computing solution that uses vehicles with idle resources as fog nodes, users can directly obtain content from fog nodes close to the user, and they no longer need to obtain content data from a relatively remote core network, through a long backhaul link. This can greatly save the waiting time between the user sending a request and being responded, thereby improving the user service quality experience.

## 6. Conclusion and Outlook

In this paper, a vehicular fog computing architecture was proposed. The vehicular fog computing architecture makes full use of the vehicles in the road and has the functions of cloud computing and fog computing. It not only solves the shortage of resources and delay in the vehicle network, but also solves the shortage of communication resources. However, due to the high mobility of the vehicle nodes, it is necessary to model the behavior of the vehicle in order to thoroughly analyze the behavior of the vehicle. Therefore, based on the previous research results of complex networks and hypergraph theory, and based on the vehicle cooperation relationship in the fog computing environment, this paper established a vehicular cooperation network model, established a more general dynamic equation, and then applied Poisson stochastic process theory. The average field method was used to analyze the evolution law of the model, and the theoretical expression of the super-degree distribution of the node was obtained. The corresponding super-degree distribution was shown in Equation (7), where *m*, *φ*, *k*1 and *k*2 are undetermined coefficients. Although the model is proposed for cooperation vehicular network in vehicular fog computing, the model can be adapted to various cooperative complex networks by adjusting parameters such as *m*, *φ*, *k*1 and *k*2, showing the universality of the model.

With the continuous deepening of theoretical research, more and more people have discovered that the intrinsic growth mechanism of actual networks shows great complexity. Accurate mathematical models can describe the common characteristics and intrinsic nature of networks. Problems such as difficulty in obtaining real data need to be solved with the help of advanced technology, and theoretical results should be applied to actual systems to provide reference for decision makers.

## Figures and Tables

**Table 1:** Table of notations.

Symbol	Definition
m0	The number of cooperation vehicles in the initial network.
k1	Number of newly added nodes in the process of forming super-edges.
K2	Number of nodes in the network during the formation of super-edges.
pi	Probability of number k1 of vehicles newly joining the vehicle cooperation network
λ1	the Poisson process of the constant.
pj	Probability of selecting the number of nodes in the network during the formation of a super-edge.
$f_{i}^{1}$	Meet and assist probability.
$d_{H}(i)$	Super-degree of the vehicle, i.
α	Harmonic parameter.
$f_{i}^{2}$	Probability that vehicle i is selected for connection.
n(t)	Total number of nodes in the network at time, t.
φ	Percentage of connections by super-degree.
